# Dynamic Assessment of Fine Motor Control and Vocalization in Parkinson Disease Through a Smartphone App: Cross-Sectional Study of Time-Severity Interaction Effects

**DOI:** 10.2196/69028

**Published:** 2025-11-13

**Authors:** Youho Myong, Seo Jung Yun, Kyudong Park, Byung-Mo Oh, Han Gil Seo

**Affiliations:** 1 Department of Biomedical Engineering, Seoul National University College of Medicine, Seoul, Republic of Korea; 2 Department of Rehabilitation Medicine, Seoul National University Hospital, 101 Daehak-no, Jongno-gu, Seoul, 03080, Republic of Korea, 82 2-2072-1659; 3 Medical Research Center, Seoul National University, Seoul, Republic of Korea; 4 Department of Human Systems Medicine, Seoul National University College of Medicine, Seoul, Republic of Korea; 5 Department of Artificial Intelligence Applications, Kwangwoon University, Seoul, Republic of Korea; 6 School of Information Convergence, Kwangwoon University, Seoul, Republic of Korea; 7 Department of Rehabilitation Medicine, Seoul National University College of Medicine, Seoul, Republic of Korea; 8 Institute on Aging, Seoul National University, Seoul, Republic of Korea

**Keywords:** Parkinson Disease, Mobile Applications, Digital Biomarkers, Voice, Motor Skills

## Abstract

**Background:**

Parkinson disease (PD) is a progressive neurodegenerative disorder characterized by motor and nonmotor symptoms that worsen over time, significantly impacting quality of life. While clinical evaluations such as the Unified Parkinson’s Disease Rating Scale (UPDRS) are standard for assessing disease severity, they offer somewhat limited temporal resolution and are susceptible to observer variability. Smartphone apps present a viable method for capturing detailed fluctuations in motor and vocal functions in real-world settings.

**Objective:**

This study aimed to use a smartphone-based app to quantitatively evaluate the interaction effect between time and disease severity on motor and vocal symptoms in individuals with PD.

**Methods:**

This was an exploratory, cross-sectional pilot study. Disease severity in persons with PD was assessed using the modified Hoehn & Yahr Scale, Voice Handicap Index, and UPDRS. We used a custom smartphone app to administer finger-tapping tasks, sustained phonation (/a/ and /i/), and rapid syllable repetition (/dadada/ and /pa-ta-ka/). The total tap counts, tap-to-tap variability, and vocal parameters (loudness, jitter, shimmer, repeat counts, and their variability) were analyzed. Each task was divided into 5 equal time frames to analyze performance changes over a short duration. Time-severity interactions were examined using linear mixed models.

**Results:**

In total, 20 persons with PD and 20 healthy adults were included in this study. Persons with PD showed worse motor and vocal performance compared to healthy adults, with higher dysrhythmia; worse jitter, shimmer, and jitter and shimmer variability; and fewer repeat counts. During finger-tapping tasks, individuals with PD showed an earlier onset of dysrhythmia than their healthy counterparts. While a higher UPDRS part III score was associated with greater finger-tapping variability, there was no significant time-severity interaction for this motor task. However, linear mixed model analysis revealed significant time-severity interaction effects for vocal tasks, including /a/ loudness (*P*=.001), /a/ jitter (*P*=.01), /a/ shimmer (*P*=.001), /i/ loudness (*P*=.001), /i/ jitter (*P*<.001), /i/ shimmer (*P*<.001), and /pa-ta-ka/-variability (*P*=.04). This indicates that individuals with higher UPDRS part III scores experienced a more rapid decline in vocal control during the assessment period. All measured smartphone-based characteristics showed a significant correlation with UPDRS part III scores, with finger-tapping variability having the strongest correlation.

**Conclusions:**

This study demonstrates that a smartphone-based assessment, conducted over just a few minutes, can detect subtle temporal changes in fine motor and vocal control. The app successfully captured the earlier onset of dysrhythmia in individuals with PD and, importantly, identified significant time-severity interaction effects in vocal performance. This suggests that such digital tools can provide sensitive, dynamic insights into symptom progression, potentially enabling more precise monitoring and timely clinical interventions for individuals with PD.

## Introduction

### Background

Parkinson disease (PD) is a progressive neurodegenerative disorder characterized by the degeneration of dopaminergic neurons in the substantia nigra, a critical region of the basal ganglia [1,2]. This neuronal loss causes a marked decrease in dopamine, a neurotransmitter essential for regulating motor control [3]. Therefore, patients with PD experience motor symptoms such as bradykinesia, rigidity, resting tremor, and postural instability, progressively impairing their ability to perform daily activities. PD is further associated with a range of nonmotor symptoms that often precede motor symptoms. These include autonomic dysfunction [4], sleep disturbances [5], neuropsychiatric symptoms [6], sensory deficits [7], and gastrointestinal issues [8]. These symptoms highlight the broad impact of PD beyond large motor impairments that complicate disease management.

Of particular relevance to this study is the impact of PD on speech and fine motor control, which are closely linked to disruptions in basal ganglia–thalamocortical circuits [1,2,9]. As the disease progresses, patients often develop hypophonia and dysarthria that significantly impair their ability to communicate effectively [10]. These symptoms, combined with motor impairments, underscore the complexity of PD and highlight the need for comprehensive diagnostic and therapeutic strategies.

Finger tapping is a widely recognized assessment tool for evaluating motor dysfunction in PD [11]. The bradykinesia and decreased motor coordination associated with PD result in reduced total tap counts, increased variability in tap frequency, and impaired rhythmicity [12,13]. Recent studies have reported dysrhythmia or increased tap-to-tap variability as the main diagnostic features of PD using smartphone app [14-16]. Voice characteristics such as loudness, jitter (frequency variability), and shimmer (amplitude variability) are often altered in persons with PD owing to the rigidity and bradykinesia of the vocal muscles [10,17]. Vocal function tasks, such as producing sustained vowel sounds (/a/, /i/) and rapid syllable repetition (/dadada/, /pa-ta-ka/), can reveal subtle changes in voice control and coordination, making them valuable for assessing dysdiadochokinesis (difficulty with rapid alternating movements) and interval variability [18,19].

Traditionally, the diagnosis and assessment of disease progression of PD have relied on clinical observations of motor symptoms [2]. However, these methods can be subjective and may vary among clinicians, making episodic assessments during clinic visits inadequate for capturing day-to-day symptom variability, particularly in the early stages when symptoms may be subtle and fluctuating [19].

In addition to clinical observations, recent advancements in diagnostic methodologies have broadened the spectrum of tools available for the diagnosis of PD. Neuroimaging techniques such as dopamine transporter single-photon emission computed tomography and neuromelanin-sensitive magnetic resonance imaging have shown utility in supporting clinical diagnosis by visualizing nigrostriatal degeneration and related pathophysiology [1]. Furthermore, genetic testing is increasingly recognized as a valuable tool, particularly in patients with early-onset or familial PD. Mutations in genes such as *LRRK2*, *GBA*, and *SNCA* have been linked to specific subtypes of PD and may inform not only diagnosis but also prognosis and therapeutic selection. Recent commentary emphasizes the importance of integrating genetic testing into routine clinical care for patients with PD [2]. These evolving diagnostic modalities complement traditional clinical assessments and support the need for objective, technology-based tools for earlier and more precise characterization of PD symptomatology.

Such technological needs have paved the way for more accessible and continuous monitoring solutions, such as smartphone-based apps [20,21]. These apps leverage the sensors and processing capabilities of smartphones to perform real-time assessments of motor and vocal functions, offering a noninvasive, continuous monitoring tool that can be used in natural environments [17]. Smartphone-based assessments enable millisecond-level quantitative evaluation of dysrhythmia without observer biases [14]. Furthermore, the integration of smartphone-based monitoring into the diagnostic process for PD offers several advantages, including frequent symptom tracking in the patient’s natural environment, providing a more comprehensive picture of disease progression [19,22]. This approach may improve diagnostic accuracy, particularly in the early stages of PD when traditional clinical assessments may miss subtle symptoms [23,24]. Furthermore, by enabling remote monitoring, these apps can enhance patient care by providing real-time feedback to clinicians and enabling timely adjustments to treatment plans [19].

### Objectives

In this study, we developed a smartphone app to monitor PD-related motor and vocal symptoms. Using this app, we evaluated the finger-tapping and voice characteristics of individuals with PD and healthy adults. This study hypothesized that individuals with more severe PD would experience earlier onset of decline in vocal and fine motor control. Therefore, this study aimed to identify the differences in vocal and fine motor control function deterioration among persons with different levels of PD severity and confirm the time-severity interaction effect on finger-tapping and vocal assessments. In doing so, the authors expect that this study investigation would provide additional insights into the time-lapse perspective on PD diagnosis and severity assessment, leading to more precise diagnostic criteria and severity stratification.

## Methods

### Study Design and Participants

This was an exploratory, cross-sectional pilot study. The inclusion criteria for healthy adults were as follows: (1) age >19 years and (2) those who voluntarily consented in writing and agreed to participate in the study. The exclusion criteria were as follows: (1) history of neurological or orthopedic conditions that could impair upper limb function, (2) history of neurological or otolaryngological conditions that might impact voice and speech, (3) presence of facial paralysis, and (4) individuals considered unsuitable due to other concurrent conditions. The inclusion criteria for patients with PD were as follows: (1) age >19 years, (2) clinical diagnosis of idiopathic PD according to the UK Parkinson’s Disease Society Brain Bank Diagnostic Criteria, and (3) modified Hoehn and Yahr (H&Y) scale stage 1‐3. The exclusion criteria were as follows: (1) history of neurological or orthopedic diseases other than PD that could affect upper limb function; (2) history of neurological or otolaryngological diseases other than PD that could affect voice and speech; (3) presence of facial paralysis due to conditions other than PD; (4) a diagnosis of PD dementia based on the Korean Montreal Cognitive Assessment (K-MoCA), with the following cutoff scores [25]: <7 points: illiterate, <13 points: education duration of 0.5 to 3 years, <16 points: education duration of 4 to 6 years, <19 points: education duration of 7 to 9 years, and <20 points: education duration of 10 years or more; (5) presence of severe dyskinesia or on-off fluctuations; and (6) any comorbid conditions deemed to make study participation difficult [25]. Participants were recruited from an outpatient clinic at Seoul National University Hospital between January 2023 and January 2024, with the last follow-up date being January 23, 2024. Evaluations for patients with PD were conducted in the “on” state, representing the peak effect of PD medication. The “on” state refers to a self-perceived period of optimal physical function following antiparkinsonian medication. In this study, most assessments were performed approximately 1 to 1.5 hours after medication intake, when participants commonly reported being in their optimal functional state.

### Ethical Considerations

The study protocol was approved by the institutional review board of Seoul National University Hospital (2211-048-1377; approved on December 16, 2022). Written informed consent was obtained from all participants. All study data were anonymized before analysis. This study was conducted in accordance with the principles of Good Clinical Practice and the Declaration of Helsinki. All participants received monetary compensation covering transportation to and from the study site. The participant pictured in this study provided written consent to publish the deidentified photograph for academic purposes.

### Experimental Design

**Figure 1.**
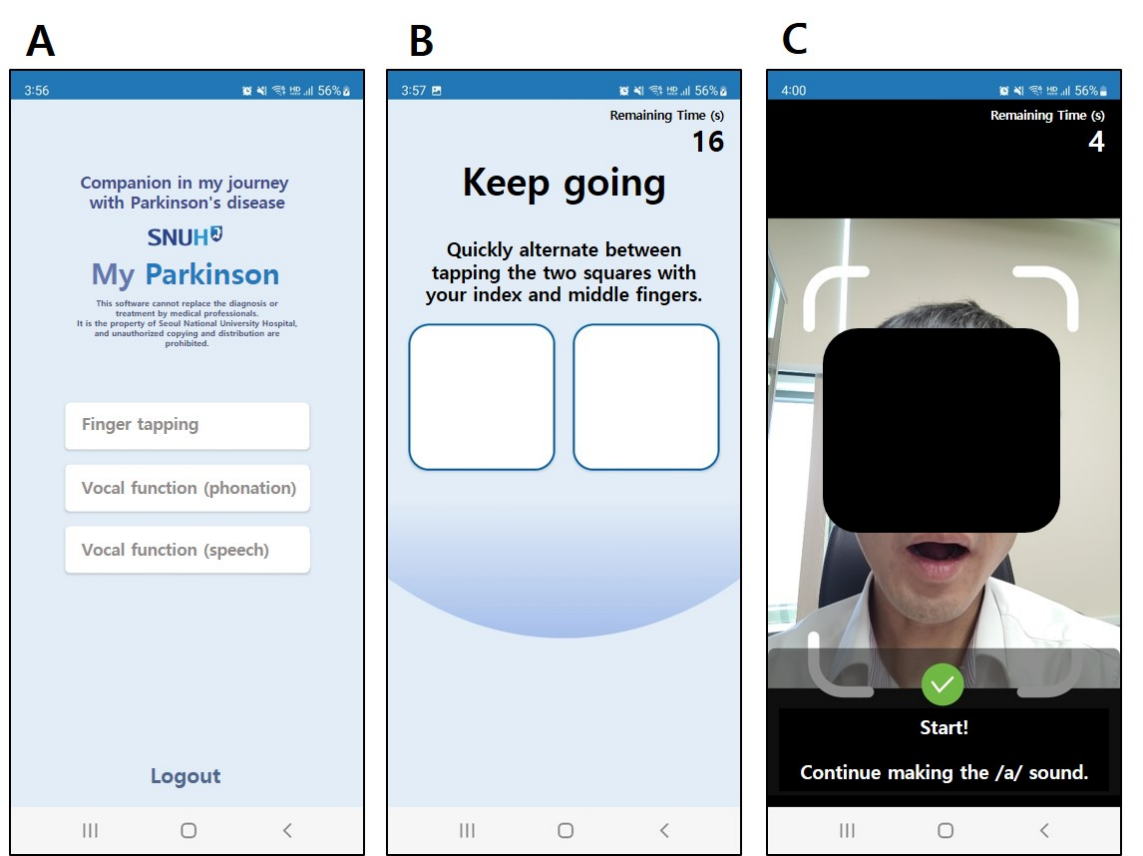
Screenshots of the smartphone app: (**A**) main menu, (**B**) finger-tapping task, and (**C**) phonation task. For the convenience of readers, the text in the figure was translated into English (originally in Korean).

An in-house smartphone app was developed to quantify the fine motor control and vocal functions (Figure 1A). The app collected data on finger-tapping and various voice parameters using the built-in touch screen, microphone, and camera of an Android (Google LLC) smartphone. Access to the app was secured using an ID and a password. During registration, participant information such as name, date of birth, sex, and year of PD diagnosis was collected. Before each assessment session, participants were prompted by the app to input the time of their most recent antiparkinsonian medication intake. The app automatically recorded the start time of each assessment, allowing calculation of the elapsed time between medication intake and task performance.

For the finger-tapping assessment, participants were instructed to alternately tap 2 squares displayed on the smartphone screen as quickly as possible using the index and middle fingers for 20 seconds (Figure 1B). Separate menus were provided for the right and left hands. The voice assessment comprised 4 tasks: 2 phonation tasks and 2 speech tasks. In the phonation tasks, participants were required to sustain the vowel sounds /a/ and /i/ for 10 seconds without interruption. Each task was performed 3 times, with a 3-second rest between trials, and the transitions between the tasks and rest periods were automated. The speech assessment included alternating motion rate and sequential motion rate tasks. The alternating motion rate tested participants’ ability to rapidly and repeatedly articulate the same phoneme, with instructions to say /dadada/ for 5 seconds. This was repeated 3 times with a 3-second interval between repetitions. The sequential motion rate evaluated the capacity to articulate different phonemes in sequence, where participants were instructed to say /pa-ta-ka/ for 5 seconds and repeat 3 times with 3-second breaks. Voice recordings were made using a smartphone microphone. To ensure consistent audio levels, the camera detected the participant’s face and initiated recordings only when the face was within a specified distance from the device (Figure 1C). If the participant moved outside the predefined range either before or during the rest period, the test did not proceed.

The participants, including both healthy adults and persons with PD, underwent a single session of finger-tapping and vocal function evaluation using a smartphone app. All evaluations were conducted in a controlled hospital environment using a Galaxy S10 (Samsung) provided by the research team, with the investigator present to ensure standardized administration. Each participant completed all assessments twice. For healthy adults, only the age and sex were recorded. The baseline characteristics of persons with PD, including age, sex, disease duration, H&Y stage, Movement Disorder Society-Unified Parkinson’s Disease Rating Scale (MDS-UPDRS) score, K-MoCA score, and Voice Handicap Index-10 (VHI-10), were collected. The MDS-UPDRS was developed to assess the impact of PD on activities of daily living and overall disease burden, allowing for the evaluation of both motor and nonmotor symptoms [26]. The MoCA assesses 8 cognitive domains to screen for cognitive impairment [27]. The VHI-10 is a self-assessment tool that measures the perceived severity of voice disorders and is categorized into functional, physical, and emotional domains [28]. It is widely used in adult patients with various types of voice disorders.

### Data Extraction

In the finger-tapping task, data were collected using a mobile app that recorded the frequency of participants’ alternating button taps on the screen. Only taps in which the participants alternated between the left and right buttons were included in the analysis. Instances in which a single button was pressed consecutively without alternating were excluded. To assess the variability in the tapping frequency, the SD of the time intervals between consecutive taps was calculated.

For voice analysis, we used the OpenSMILE for Python toolkit version 2.5.0 (audEERING®) [29] to extract specific acoustic features from the eGeMAPSv02 feature set [30]. The extracted features included loudness, jitter, and shimmer, which are commonly used in speech analysis to quantify variations in voice quality. To assess the variability of jitter and shimmer, the coefficient of variation, a normalized measure of variability that represents the ratio of the SD to the mean, was calculated. This approach allowed quantification of the relative variability in these acoustic features, providing insights into the participants’ voice modulation patterns. In addition, during the speech tasks in which participants produced the /dadada/ and /pa-ta-ka/ sounds, variability in the frequency of speech production was calculated as the SD of the time intervals between consecutive sounds. This measure helped to evaluate the consistency of sound production, offering further insight into the participants’ speech motor control and temporal coordination during these tasks.

### Statistical Analysis

Data are expressed as means and SDs for continuous variables and as frequencies (percentages) for categorical variables. Each recorded entry of finger-tapping (20 s on each side) and vocal tasks (10 s for phonation and 5 s for speech) was divided into 5 equal time frames (TFs), with the first TF being TF1, and the last being TF5 (ie, for finger tapping: TF1=0 s-4 s, TF2=4 s-8 s, TF3=8 s-12 s, TF4=12 s-16 s, and TF5=16 s-20 s; for phonation: TF1=0 s-2 s, TF2=2 s-4 s, TF3=4 s-6 s, TF4=6 s-8 s, and TF5=8 s-10 s; and for speech: TF1=0 s-1 s, TF2=1 s-2 s, TF3=2 s-3 s, TF4=3 s-4 s, and TF5=4 s-5 s). ANOVA was conducted to determine whether there were significant differences between the different TFs and groups [31]. A linear mixed model (LMM) was applied to examine the time-group and time-severity interaction effects within each data group [32]. Statistical significance was set at *P*<.05. All statistical analyses were performed using SPSS (version 26.0; IBM Corp).

## Results

### Study Participants

In total, 20 individuals with PD and 20 healthy adults without a history of neurological disorders were enrolled in this study. The patient group comprised 85% (17/20) female participants with a mean age of 68.30 (SD 6.30) years. The H&Y scale was distributed as follows: 1.5 (4/20, 20%), 2 (5/20, 25%), 2.5 (7/20, 35%), and 3 (4/20, 20%). The mean MDS-UPDRS part III score was 22.10 (SD 10.64), the mean VHI-10 12.05 (SD 9.59), and the mean K-MoCA 25.25 (SD 2.40). The healthy adults group comprised 50% (10/20) female participants with a mean age of 33.55 (SD 7.85) years. The demographic information of the study participants is presented in Table 1.

**Table 1.** Demographic characteristics (N=20 for each group).

	Persons with PD^a^	Healthy adults
Age (years), mean (SD)	68.30 (6.30)	33.55 (7.85)
Sex (female), % (n/N)	85 (17/20)	50 (10/20)
H&Y^b^ scale, % (n/N)		
1.5	20 (4/20)	N/A^c^
2	25 (5/20)	N/A
2.5	35 (7/20)	N/A
3	20 (4/20)	N/A
Disease duration (years), mean (SD)	8.90 (3.78)	N/A
UPDRS^d^ total, mean (SD)	50.90 (16.00)	N/A
UPDRS part III, mean (SD)	22.10 (10.64)	N/A
VHI-10^e^, mean (SD)	12.05 (9.59)	N/A
K-MoCA^f^, mean (SD)	25.25 (2.40)	N/A

a PD: Parkinson disease.

b H&Y: Hoehn and Yahr.

c Not applicable.

d UPDRS: Unified Parkinson’s Disease Rating Scale.

e VHI-10: Voice Handicap Index 10.

f K-MoCA: Korean Montreal Cognitive Assessment.

### Overall Characteristics of Finger Tapping

The patient group demonstrated a lower total number of taps (mean 76.250, SD 35.932 vs mean 199.263, SD 32.600; *P*<.001) and a notably higher tap frequency variability (mean 0.158, SD 0.144 vs mean 0.0288, SD 0.0101; *P* <.001) than the healthy adult group. This phenomenon was consistently observed across all TFs when the total experimental time was evenly divided into 5 segments (TF1-TF5). The results of the mean total number of taps and tap-to-tap variability are presented in Table 2.

**Table 2.** Time frame (TF)–wise analysis of tap-to-tap variability.

	Healthy adults, mean (SD)	Persons with PD^a^, mean (SD)	*t* value (df)	*P* value
Total taps	199.263 (32.600)	76.250 (35.932)	22.678 (38)	<.001
Mean variability	0.0288 (0.0101)	0.158 (0.144)	–8.017 (38)	<.001
TF 1	0.0238 (0.0100)	0.101 (0.0803)	–8.563 (38)	<.001
TF 2	0.0238 (0.0100)	0.113 (0.0744)	–10.657 (38)	<.001
TF 3	0.0258 (0.0109)	0.130 (0.0920)	–10.067 (38)	<.001
TF 4	0.0284 (0.0128)	0.134 (0.0860)	–10.897 (38)	<.001
TF 5	0.0323 (0.0124)	0.143 (0.0881)	–11.303 (38)	<.001

a PD: Parkinson disease.

### Time-Group Interaction Effects on Finger Tapping

Both groups experienced increasing tap-to-tap variability as the TF progressed (*P*<.001). Furthermore, the LMM analysis revealed a significant time-by-group interaction during the finger-tapping task (*P*=.02), implying that the effect of time on increasing tap-to-tap variability was experienced differently by persons with PD and healthy adults (Table 3).

**Table 3.** Interaction effect of time and group on finger tapping.

	Coefficient (SE; 95% CI)	*t* value (df)	*P* value
Intercept	0.021 (0.012; –0.002 to 0.044)	1.815 (38)	.07
Time frame	0.072 (0.017; 0.040 to 0.105)	4.372 (38)	<.001
Group	0.002 (0.003; –0.003 to 0.007)	0.683 (38)	.50
Time frame: group	0.009 (0.004; 0.002 to 0.016)	2.388 (38)	.02

The positive coefficient in the TF:group row in Table 3 indicates that the patient group exhibited greater early-onset variability compared with the control group. This finding was further supported by the ANOVA and post hoc analysis of interval variability.

To assess whether the tapping intervals in both groups remained consistent or if fatigue was observed, an ANOVA was conducted on the interval variability across TF1 to TF5, which revealed a significant difference in the mean interval variability across the 5 TFs (*P*=.001 for healthy adults and *P=*.02 for persons with PD). Post hoc analyses found that while the interval variability of the control group significantly increased starting from TF3 (*P=*.01), that of the patient group did so earlier, starting from TF2 (*P=*.05). Together with the findings in Table 3, this implies that there was a meaningful interaction between time and group. More specifically, the patient group started to experience increasing variability earlier than the controls. The results of the ANOVA and post hoc analyses are described in (Tables S1-S4 in Multimedia Appendix 1).

### Interaction of Time and Disease Severity on Finger Tapping in Persons With PD

While a higher UPDRS part III score indicated higher interval variability (*P*=.001), there was no significant interaction effect between time and disease severity (Table 4).

**Table 4.** Interaction effect between time and disease severity.

	Coefficient (SE; 95% CI)	*t* value (df)	*P* value
Intercept	0.0290 (0.021312; –0.0129 to 0.0709)	1.359 (19)	.18
Time frame	0.0112 (0.006426; –0.0015 to 0.0238)	1.739 (19)	.08
UPDRS^a^ III	0.00291 (0.000869; 0.0012 to 0.0046)	3.352 (19)	.001
Time frame: UPDRS part III	–0.000376 (0.000262; –0.00055 to 0.00048)	–0.143 (19)	.89

a UPDRS part III: Unified Parkinson’s Disease Rating Scale part III.

### Overall Characteristics of Voice

The persons with PD group demonstrated significantly louder volumes (*P<*.001 for both /a/ and /i/) and higher mean jitter (*P<*.001 for /a/ and *P=*.006 for /i/), jitter variability (*P<*.001 for both /a/ and /i/), mean shimmer (*P=*.008 for /a/), and shimmer variability (*P<*.001 for both /a/ and /i/) in sustained phonation. For repeating speech tasks (/dadada/ and /pa-ta-ka/ sounds), the persons with PD group showed lower total repeats (*P<*.001 for both /dadada/ and /pa-ta-ka/), with elevated interval variability (*P<*.001 for /dadada/). Voice characteristics are presented in Table 5. There was no significant time-group interaction in the voice analysis (Table S5 in Multimedia Appendix 1).

**Table 5.** Group comparison on voice characteristics.

	Healthy adults, mean (SD)	Persons with PD^a^, mean (SD)	*t* value (df)	*P* value
Sustained phonation				
Vowel /a/				
Loudness	1.573 (0.735)	2.051 (0.844)	–4.514 (38)	<.001
Mean jitter	0.0074 (0.00521)	0.151 (0.0174)	–4.647 (38)	<.001
Jitter variability	1.930 (1.583)	3.181 (1.959)	–5.180 (38)	<.001
Mean shimmer	0.477 (0.196)	0.578 (0.332)	–2.692 (38)	.008
Shimmer variability	0.737 (0.303)	0.971 (0.337)	–5.446 (38)	<.001
Vowel /i/				
Loudness	1.082 (0.503)	1.302 (0.519)	–3.225 (38)	.001
Mean jitter	0.0071 (0.00583)	0.0103 (0.0105)	–2.773 (38)	.006
Jitter variability	2.011 (1.960)	3.673 (2.420)	–5.608 (38)	<.001
Mean shimmer	0.402 (0.190)	0.417 (0.246)	–0.497 (38)	.62
Shimmer variability	0.766 (0.352)	1.071 (0.452)	–5.577 (38)	<.001
Speech				
/dadada/				
Repeat count	40.067 (4.623)	36.225 (5.329)	5.791 (38)	<.001
Interval variability	1.931 (0.874)	3.705 (2.755)	–6.325 (38)	<.001
/pa-ta-ka/				
Repeat count	41.010 (7.011)	37.442 (5.475)	4.089 (38)	<.001
Interval variability	4.970 (10.808)	5.087 (3.938)	–0.101 (38)	.91

a PD: Parkinson disease.

### Interaction of Time and Disease Severity on Voice Characteristics in Persons With PD

A significant time-severity interaction effect was observed in many aspects of voice analysis. Specifically, time and UPDRS part III interaction effects were present in /a/ loudness (*P*=.001), /a/ jitter (*P*=.01), /a/ shimmer (*P*=.001), /i/ loudness (*P*=.001), /i/ jitter (*P*<.001), /i/ shimmer (*P*<.001), and /pa-ta-ka/-variability (*P*=.04). These results suggest that individuals with different disease severities exhibit significantly different performance trajectories over time. Participants with higher UPDRS part III scores showed greater declines in vocal and fine motor control over time, reflected by larger increases in jitter and shimmer and higher repeat variability in later time frames compared with those with lower scores. It is noteworthy that the time-disease severity interaction effect was significant in dependent variables where the independent time or independent disease severity effect was not statistically significant. This will be explored in greater depth in the Discussion section. The detailed results of the LMM analysis are described in Table 6.

**Table 6.** Interaction effect between time and Unified Parkinson’s Disease Rating Scale part III (UPDRS part III) score in persons with Parkinson disease.

Variable		Coefficient (SE)	*t* value (df)	*P* value
Sustained phonation				
Vowel /a/				
Loudness				
Intercept		2.319 (0.190)	12.203 (19)	<.001
TF^a^		0.0825 (0.0573)	1.440 (19)	.15
UPDRS		0.00123 (0.00789)	0.155 (19)	.88
TF:UPDRS		–0.00830 (0.00238)	–3.489 (19)	.001
Jitter				
Intercept		0.00929 (0.00330)	2.820 (19)	.005
TF		–0.000456 (0.000994)	–0.459 (19)	.65
UPDRS		–0.000111 (0.000137)	–0.808 (19)	.42
TF:UPDRS		0.000106 (0.0000412)	2.578 (19)	.01
Shimmer				
Intercept		0.395 (0.0790)	5.001 (19)	<.001
TF		–0.0183 (0.0238)	–0.769 (19)	.44
UPDRS		–0.000815 (0.00328)	–0.249 (19)	.80
TF:UPDRS		0.00338 (0.000989)	3.416 (19)	.001
Vowel /i/				
Loudness				
Intercept		1.341 (0.118)	11.363 (19)	<.001
TF		0.0787 (0.0356)	2.211 (19)	.03
UPDRS		0.00238 (0.00481)	0.495 (19)	.62
TF:UPDRS		–0.00492 (0.00145)	–3.389 (19)	.001
Jitter				
Intercept		0.0112 (0.00285)	3.925 (19)	<.001
TF		–0.00232 (0.000860)	–2.700 (19)	.007
UPDRS		–0.000357 (0.000116)	–3.068 (19)	.002
TF:UPDRS		0.000196 (0.0000351)	5.602 (19)	<.001
Shimmer				
Intercept		0.393 (0.0674)	5.831 (19)	<.001
TF		–0.0470 (0.0203)	–2.310 (19)	.02
UPDRS		–0.00823 (0.00275)	–2.993 (19)	.003
TF:UPDRS		0.00498 (0.000829)	6.013 (19)	<.001
Speech				
/dadada/-variability				
Intercept		1.474 (0.441)	3.343 (19)	.001
TF		–0.0433 (0.133)	–0.326 (19)	.74
UPDRS		0.0462 (0.0180)	2.570 (19)	.01
TF:UPDRS		0.00307 (0.00542)	0.566 (19)	.57
/pataka/-variability				
Intercept		0.424 (0.746)	0.569 (19)	.57
TF		0.724 (0.225)	3.220 (19)	.001
UPDRS		0.0961 (0.0304)	3.164 (19)	.002
TF:UPDRS		–0.0194 (0.00916)	–2.117 (19)	.04

a TF: time frame.

### Correlation and Linear Regression Analyses of Disease Severity on Finger Tapping and Voice Characteristics

All voice characteristics and finger tapping were significantly correlated with the UPDRS part III scores. Linear regression analysis revealed that finger tapping had the strongest correlation with the UPDRS part III score (*r*=0.35; *R*^2^=0.123; *P*<.001). The results of the correlation and linear regression analyses are presented in Table 7.

**Table 7.** Correlation and linear regression analysis of disease severity on finger tapping and voice characteristics.

	Correlation coefficient (*r*)	*R* ^2^ ^a^	*P* value
Finger tapping	0.35	0.123	<.001
Sustained phonation			
Vowel /a/			
Loudness	–0.27	0.073	<.001
Jitter	0.14	0.02	<.001
Shimmer	0.25	0.065	<.001
Vowel /i/			
Loudness	–0.24	0.056	<.001
Jitter	0.18	0.032	<.001
Shimmer	0.21	0.046	<.001
Speech			
/dadada/-variability	0.28	0.081	<.001
/pa-ta-ka/-variability	0.12	0.014	.004

a *R*2: coefficient of determination in linear regression analysis.

## Discussion

### Principal Findings

This study investigated chronological differences in vocal and fine motor control deterioration among individuals with varying levels of PD severity, with the goal of discovering the interaction effect between time and disease severity on finger-tapping and vocal assessments. Voice analysis demonstrated significant time-severity interaction effects in many aspects. Persons with higher UPDRS part III scores showed greater increases in /a/-loudness, /a/-jitter, /a/-shimmer, /e/-loudness, /e/-jitter, /e/-shimmer, and /pa-ta-ka/-variability. Such time-severity interaction effects were present even in the dependent variables where the independent time or independent disease severity effect was not statistically significant. Finger-tapping performance was significantly affected only by UPDRS part III scores, with no evident time-severity interaction.

In recent years, there has been growing interest in using smartphone-based apps to provide objective and continuous monitoring of PD symptoms [13,17,19-21,23,24]. Unlike traditional assessments, a smartphone app can continuously and quantitatively monitor finger-tapping performance and detect subtle changes in motor function that might not be evident during a physical examination [33]. By capturing tap-to-tap variability in finger tapping down to milliseconds and analyzing the data across 5 TFs, it became possible to examine both the time effect and the time-group interaction effect in individuals with PD and healthy adult groups. This continuous data collection allows for the detection of variability and subtle symptoms that might otherwise go unnoticed, thereby providing a more comprehensive understanding of a patient’s condition over time [19]. The group effect between the patient and healthy adult groups was evident, as shown in Table 2. Further intriguing was the time-group interaction effect observed in finger tapping, as shown in Table 3 and Tables S1-S4 in Multimedia Appendix 1. This suggests that the temporal worsening of dysrhythmia develops differently, even among persons with PD, depending on the disease severity.

In addition to motor symptoms, PD also affects vocal function, presenting in symptoms such as hypophonia (reduced speech volume) and dysarthria (difficulty articulating speech) [6,17,34]. These symptoms are linked to the rigidity and bradykinesia of the vocal muscles, which are also controlled by the basal ganglia–thalamocortical circuits [35]. In this study, vocal characteristics such as loudness, jitter, and shimmer were significantly impaired in persons with PD compared to healthy adults. The increased interval variability observed during fast-alternating vocalization tasks (/dadada/ and /pa-ta-ka/) further indicated impaired articulatory control in patients with PD [19].

Statistical analysis revealed significant time-disease severity interaction effects for nearly all the voice characteristics examined. Notably, this interaction effect was observed even when the individual effects of time or UPDRS part III scores on the voice characteristics (eg, loudness, jitter, and shimmer) were not significant. This phenomenon highlights an important insight often seen in statistical analysis: the main effects may be non-significant, yet the interaction effect can still be meaningful. In such scenarios, while the UPDRS part III scores may not show a significant impact on vocal function when averaged across all TFs, their effect may vary significantly across different periods. For instance, a patient might experience minimal vocal changes during sustained phonation in the early stages of the disease. However, as the condition progresses, the impact on vocal changes could become more pronounced [35]. This underscores the importance of considering interaction effects within the model, as they can reveal critical dynamics that are not captured by the main effects alone.

The time-disease severity interaction within the patient group further suggests that the progression of vocal symptoms varies according to disease severity over time, emphasizing the need for dynamic, longitudinal evaluations of vocal function in individuals with PD. As PD advances, the impact on vocal symptoms fluctuates depending on the severity at different stages, with those experiencing mild disease severity initially showing minimal changes, which may later intensify as the disease progresses [23,35,36]. This variability highlights the need for the regular monitoring of vocal function to allow timely adjustments in treatment strategies. Longitudinal assessments are essential for capturing the nuanced progression of symptoms and tailoring interventions to meet the evolving needs of patients [19,24,35].

The time-severity interaction effect observed in vocal function suggests that individuals with higher UPDRS part III scores experience greater vocal fatigue over time. Increased inhibition of the thalamus leads to a significant reduction in the ability of the motor cortex to generate and sustain precise, coordinated movements required for normal speech. Therefore, these individuals experience greater vocal fatigue, characterized by earlier and more severe reductions in vocal loudness and increased variability in pitch (jitter) and amplitude (shimmer). Over time, the continued loss of dopamine exacerbates these issues, resulting in a progressive decline in vocal function. While the correlation coefficients in Table 7 are modest, this may reflect the difference in temporal resolution and scope between app-based metrics and clinical rating scales. Smartphone measures capture specific, moment-to-moment performance, whereas clinical scores aggregate broader symptom impressions over time.

The earlier and greater exacerbation of dysrhythmia and vocal control deficits in individuals with higher disease severity reflects progressive degeneration of the basal ganglia–thalamocortical circuits [9,37]. Finger-tapping dysrhythmia is linked to the dysfunction of direct and indirect pathways, particularly those involving the degeneration of dopaminergic neurons in the substantia nigra pars compacta. As the substantia nigra pars compacta dopaminergic output to the striatum decreases with advancing disease, the inhibitory-excitatory balance within the striatonigral (direct) and striatopallidal (indirect) pathways is disrupted [16]. This imbalance exacerbates motor dysrhythmia, especially in higher-severity cases, due to increased subthalamic nucleus burst firing, which contributes to abnormal motor rhythms [14].

Conversely, vocal control deficits, which are more dependent on sustained muscle activity and coordination, are further compromised by advanced PD owing to greater degeneration in the pathways responsible for fine motor control, particularly the hyperdirect cortico-subthalamic pathway [22,24]. This pathway plays a crucial role in coordinating the speech muscles. In individuals with higher disease severity, the progressive loss of dopaminergic input leads to earlier fatigue and diminished motor precision, resulting in worsened vocal control [9].

Importantly, the strength of smartphone-based assessment lies not only in its accessibility but also in its capacity to quantify time-sensitive symptom trajectories with high granularity. Although this study did not observe a significant time-severity interaction in fine motor control (finger tapping), such effects were clearly evident in vocal characteristics. This discrepancy highlights the potential of vocal metrics as sensitive indicators of progression and suggests that certain domains may respond differently to disease burden over time. The ability to detect these nuanced changes through a smartphone app underscores its utility in capturing progression patterns that may elude conventional assessments, reaffirming the clinical value of mobile technology in PD monitoring.

Beyond the interpretation of statistical findings, this study contributes meaningfully toward broader clinical goals in the management of PD. By demonstrating that a smartphone-based assessment can detect time-severity interaction effects in both fine motor and vocal symptoms, this study supports the feasibility of dynamic, real-world monitoring of disease progression. Furthermore, the ability to capture subtle, early-onset symptom fluctuations using accessible digital tools aligns with the long-term objective of improving diagnostic accuracy, particularly in the early or prodromal stages of the disease. Ultimately, by facilitating more personalized, timely, and continuous monitoring, this approach holds the potential to enhance patient care through earlier interventions and more responsive treatment strategies.

### Limitations

This study has several limitations. First, the healthy adult group and the persons with PD group were not adequately matched for age and sex, and the demographics of the control group did not accurately represent those of the general population with PD. Future studies should ensure better age-sex matching. However, it is important to note that the significant results observed in both the group effect and within-group time-disease severity analyses were obtained regardless of this mismatch. Second, although smartphone monitoring is useful for collecting real-world data, in this study, data collection through the app was conducted in a controlled research setting rather than in a natural environment. Future research should explore the use of smartphone apps in home settings to better capture real-world data in persons with PD. Finally, the findings of this study warrant large-scale research to evaluate the diagnostic accuracy and reliability of smartphone apps. While this study focused only on finger-tapping and voice characteristics, future research should include other variables such as gait functions, blinking, and natural language analysis.

### Conclusions

This study highlights the potential of smartphone-based time-lapse assessment of symptoms to discern disease severity in persons with PD. With only a few minutes of dynamic assessment, this approach can detect subtle temporal fluctuations in dysrhythmia and vocal control deficits, providing novel insights into the severity of PD.

## Supplementary material

10.2196/69028 Multimedia Appendix 1 ANOVA and *post-hoc* analyses of voice characteristics and finger-tapping results.

## Abbreviations

H&Y: Hoehn and Yahr
K-MoCA: Korean Montreal Cognitive Assessment
LMM: linear mixed model
MDS: Movement Disorder Society
PD: Parkinson disease
TF: time frame
UPDRS: Unified Parkinson’s Disease Rating Scale
VHI-10: Voice Handicap Index-10
