# Supplementary material for: Dynamic Assessment of Fine Motor Control and Vocalization in Parkinson Disease Through a Smartphone App: Cross-Sectional Study of Time-Severity Interaction Effects
Source: JMIR Mhealth Uhealth. 2025 Nov 13;13:e69028. doi: 10.2196/69028 (PMC12614394; doi:10.2196/69028)
Supplement: Multimedia Appendix 1 [file mhealth-v13-e69028-s001.docx]

**Supplementary Table 1. Interval Variability ANOVA - Control**

| **Timeframe** | **Mean** | **Standard Deviation** | **F** | ***P-*value** |
| --- | --- | --- | --- | --- |
| **TF1** | 0.023848 | 0.010040 | 5.090 | .001 |
| **TF2** | 0.023772 | 0.010007 |  |  |
| **TF3** | 0.025842 | 0.010851 |  |  |
| **TF4** | 0.028379 | 0.012778 |  |  |
| **TF5** | 0.030231 | 0.012374 |  |  |

**Supplementary Table 2. Interval Variability ANOVA - Patients**

| **Timeframe** | **Mean** | **Standard Deviation** | **F** | ***P-*value** |
| --- | --- | --- | --- | --- |
| **TF1** | 0.101390 | 0.080371 | 3.137 | .015 |
| **TF2** | 0.113223 | 0.074401 |  |  |
| **TF3** | 0.130119 | 0.092010 |  |  |
| **TF4** | 0.134251 | 0.085957 |  |  |
| **TF5** | 0.142718 | 0.088146 |  |  |

**Supplementary Table 3. post-hoc Analysis of Interval Variability ANOVA - Control**

| **TF (A)** | **TF (B)** | **Diff. in Means (A-B)** | ***P-*value** | **95% CI** | |
| --- | --- | --- | --- | --- | --- |
|  |  |  |  | Lower | Upper |
| TF5 | **TF1** | **0.006383138** | **.013** | **0.00086823** | **0.011898** |
|  | **TF2** | **0.006459038** | **.011** | **0.00094413** | **0.011974** |
|  | TF3 | 0.0043887 | .197 | -0.0011262 | 0.0099036 |
|  | TF4 | 0.001852075 | .897 | -0.0036628 | 0.0073670 |

**Supplementary Table 4. post-hoc Analysis of Interval Variability ANOVA - Patients**

| **TF (A)** | **TF (B)** | **Diff. in Means (A-B)** | ***P-*value** | **95% CI** | |
| --- | --- | --- | --- | --- | --- |
|  |  |  |  | Lower | Upper |
| TF5 | **TF1** | **0.041328** | **.050** | **0.000013840** | **0.082642** |
|  | TF2 | 0.029495 | .305 | -0.011949 | 0.070939 |
|  | TF3 | 0.012599 | .926 | -0.028714 | 0.053913 |
|  | TF4 | 0.0084673 | .982 | -0.032846 | 0.049781 |

**Supplementary Table 5. Interaction Effect between Time and Group – Voice Analysis**

|  | **Variable** | **Coefficient** | **Std. Error** | **t-value** | ***P*-value** |
| --- | --- | --- | --- | --- | --- |
| **A - loudness** | Intercept | 1.840 | 0.163 | 11.294 | .000 |
|  | **Timeframe** | 0.506 | 0.230 | 2.204 | .028 |
|  | **Group** | -0.090 | 0.029 | -3.112 | .002 |
|  | TF:Group | -0.012 | 0.041 | -0.300 | .764 |
|  |  |  |  |  |  |
| **A - jitter** | Intercept | 0.004 | 0.001 | 2.848 | .004 |
|  | Timeframe | 0.003 | 0.002 | 1.336 | .182 |
|  | Group | 0.001 | 0.001 | 1.549 | .121 |
|  | TF:Group | 0.001 | 0.001 | 1.130 | .259 |
|  |  |  |  |  |  |
| **A - shimmer** | Intercept | 0.379 | 0.034 | 11.149 | .000 |
|  | Timeframe | -0.004 | 0.047 | -0.089 | .929 |
|  | Group | 0.026 | 0.015 | 1.672 | .094 |
|  | TF:Group | 0.030 | 0.021 | 1.400 | .162 |
|  |  |  |  |  |  |
| **E - loudness** | Intercept | 1.273 | 0.107 | 11.944 | .000 |
|  | Timeframe | 0.120 | 0.150 | 0.800 | .423 |
|  | Group | -0.029 | 0.017 | -1.702 | .089 |
|  | TF:Group | -0.001 | 0.024 | -0.032 | .974 |
|  |  |  |  |  |  |
| **E - jitter** | Intercept | 0.005 | 0.001 | 3.338 | .001 |
|  | Timeframe | -0.002 | 0.002 | -0.810 | .418 |
|  | Group | 0.000 | 0.001 | 0.750 | .453 |
|  | TF:Group | 0.002 | 0.001 | 1.741 | .082 |
|  |  |  |  |  |  |
| **E - shimmer** | Intercept | 0.326 | 0.040 | 8.191 | .000 |
|  | Timeframe | -0.114 | 0.055 | -2.063 | .039 |
|  | Group | 0.021 | 0.017 | 1.246 | .213 |
|  | TF:Group | 0.042 | 0.024 | 1.757 | .079 |
|  |  |  |  |  |  |
| **dadada - variability** | Intercept | 1.576 | 0.227 | 6.947 | <.001 |
|  | Timeframe | 0.918 | 0.316 | 2.908 | .004 |
|  | Group | -0.009 | 0.045 | -0.190 | .849 |
|  | TF:Group | 0.034 | 0.062 | 0.547 | .584 |
|  |  |  |  |  |  |
| **pataka - variability** | Intercept | 1.713 | 0.422 | 4.061 | <.001 |
|  | Timeframe | 0.822 | 0.577 | 1.423 | .155 |
|  | Group | 0.244 | 0.138 | 1.772 | .076 |
|  | TF:Group | 0.058 | 0.190 | 0.306 | .760 |
